# Influence of traffic accessibility on land use based on Landsat imagery and internet map: A case study of the Pearl River Delta urban agglomeration

**DOI:** 10.1371/journal.pone.0224136

**Published:** 2019-12-05

**Authors:** Yongwei Liu, Xiaoshu Cao, Jianbin Xu, Tao Li

**Affiliations:** 1 School of Business, Ludong University, Yantai, Shandong, China; 2 School of Geography Science and Planning, Sun Yat-sen University, Guangzhou, Guangdong, China; 3 Institute of Transport Geography and Spatial Planning, Shaanxi Normal University, Xi’an, Shaanxi, China; Tongii University, CHINA

## Abstract

Taking the Pearl River Delta urban agglomeration, with its rapid economic and social development and dramatic changes in land use, as an example with which to conduct this research, this paper analyzes the influence of traffic accessibility on land use and socioeconomic development by using Internet maps, points of interest (POI), and data about land use and socioeconomic development. The results of this study are as follows: (1) the calculation results of traffic accessibility can reflect the real situation because spatial distribution has an obvious feature of circle-like structure, and the development level of each township has high consistency; (2) major change characteristics in land use are that cultivated and forest lands are decreasing, and construction land is increasing, and it is obvious that the increase of construction land is featured by spatial autocorrelation and clusters of construction areas along the city borders; (3) traffic accessibility has a significant impact on land use status and changes and socioeconomic development, while the number of facilities has weak impact on land use, which is a reflection of advanced infrastructure in the urban agglomeration; and (4) the construction of transport infrastructure promotes the transformation of land use from water to construction land, therefore, more emphasis should be placed on protecting the river system in future infrastructure construction so as to improve the ecological benefits in the river basin.

## Introduction

There is a close interaction between transportation and land use because improving traffic accessibility could advance changes in land use and, at the same time, changes in land use could promote the development of regional traffic activities and increase transportation demands[1]. The concept of traffic accessibility is regarded as the basis for analyzing the interaction between transportation and land use[2]. The role of transportation infrastructure construction becoming more and more important, part research foucs on the road itself such as method of increase the service life of highway pavements, identified locations with a high probability of strong crosswind, and the potential effects of autonomous vehicles on transportation infrastructure[3–5], other foucs on the influence and effects[6, 7]. Correlational studies show that traffic accessibility could have an important role in land use changes and regional development, whether in developed metropolitan areas[8] and urban agglomerations[9], or in mountain areas[10, 11] and forest areas[12–14]. In addition, field surveys show that there is obvious correlation between traffic accessibility and the living standards of residents[15].

Research on the influence of traffic accessibility on land use mainly focuses on three parts, as follows. First, the influence of different road types such as railways and highways on land use[16–21]. Luo’s (2009) research on Nanjing, China shows that if land is near a railway, it hinders the development of urban land[20]. Xie’s (2010) research on the Twin Cities (Minneapolis and Saint Paul) showed that railways could advance residential development[17], while Conway (2005) indicated that accessibility of highways in New Jersey is significantly related to the development of new cities[21]. Second, the influence of traffic accessibility on land use in various zones, such as urban areas and rural areas. The influence of traffic accessibility on land use focuses on construction land[9, 22–28]. Ju (2016), using Geodetector, studied the influence of different factors on urban expansion, providing a new perspective to analyze the interaction between various influencing factors[25]. Zhang (2013) used four landscape metrics to show characteristics of urban growth, pointing out that distance from national or provincial roads would affect urban patterns and, as urbanization develops, weaken the influence of urban centers on urban expansion[9]. The influence of traffic accessibility on relatively economically backward areas, such as rural and forest areas, focuses on agricultural and forest land[10–13, 29–32]. Castella (2005), from the perspective of traffic accessibility to rural areas, indicates that village location is highly correlated with road networks and land use, and it is also related to poverty and development potential[11]. Research by Nagendra (2003) also showed that deforestation is becoming a more common phenomenon in areas with advanced transportation, but the degree of influence varies from time to time[32]. Third, the entire regional study is mainly focused on the influence on network characteristics[33–35], taking into account gradient analysis, in both urban and rural areas, of accessibility to urban centers[36–40]. Brinkmann (2012) conducted a systematic study on land use changes, the urbanization process, arable-land expansion, derelict-land development, and the deforestation process driven by agroecological-system infrastructures in four West African cities[39]. Reimets (2015) selected three main outlet roads of Tallinn, the capital of Estonia, to calculate landscape gradient metrics in order to carry out research; results showed that fragmentation of the whole landscape would be reduced as distance to roads increases, and that the degree of fragmentation caused by distance to cities is lower than that caused by the distance to roads. At the same time, the urbanization process in the suburbs does not develop in a symmetric way on both sides of the road[36]. With the help of the roadless volume (RV) index, Fu (2010) indicated that the area, and the amount of forest land and arable land, are reduced in areas with many interference factors, while area and amount are increased in areas with fewer interference factors. If the areas of urban land are increased, road networks would change the landscape pattern around the roads[40].

Nowadays, most studies are on the interaction between traffic accessibility and land use, while research on urban regions is mainly focused on the influence of traffic accessibility on certain types of land use, especially construction land. Studies on rural regions are mainly focused on studying ecological land and land for landscape ecology, rarely involving transportation, land use, and social economy. Therefore, it is necessary to optimize the quantitative calculation of traffic accessibility and to study more replicable quantitative accessibility analysis methods based on objective data to conduct comprehensive analysis on transportation, land use, and regional development. Traffic accessibility, the mediating variable that affects the development of urbanization and the social economy, plays an important role in reshaping the pattern of regional land use. Thus, it is significant to study the influence of traffic accessibility on land use, and to master the characteristics and mechanisms of land use changes in urban agglomerations. Internet map contains the detailed information such as highways entrances and exits, road connections and community road pavement, reflect the real situation better when calculate the travel time and distance, therefore, Internet map is significance method in accessibility research. Meanwhile, considering that there are few studies focusing on townships in urban agglomerations, the research in this paper focuses on the weak parts to conduct the survey so as to discuss replicable quantitative calculation analysis for traffic accessibility. With the Pearl River Delta as the research region, the study was conducted to analyze 581 townships by using Internet maps, which is the most typical and representative type of research.

## Materials and methods

### Study area

**Fig 1:**
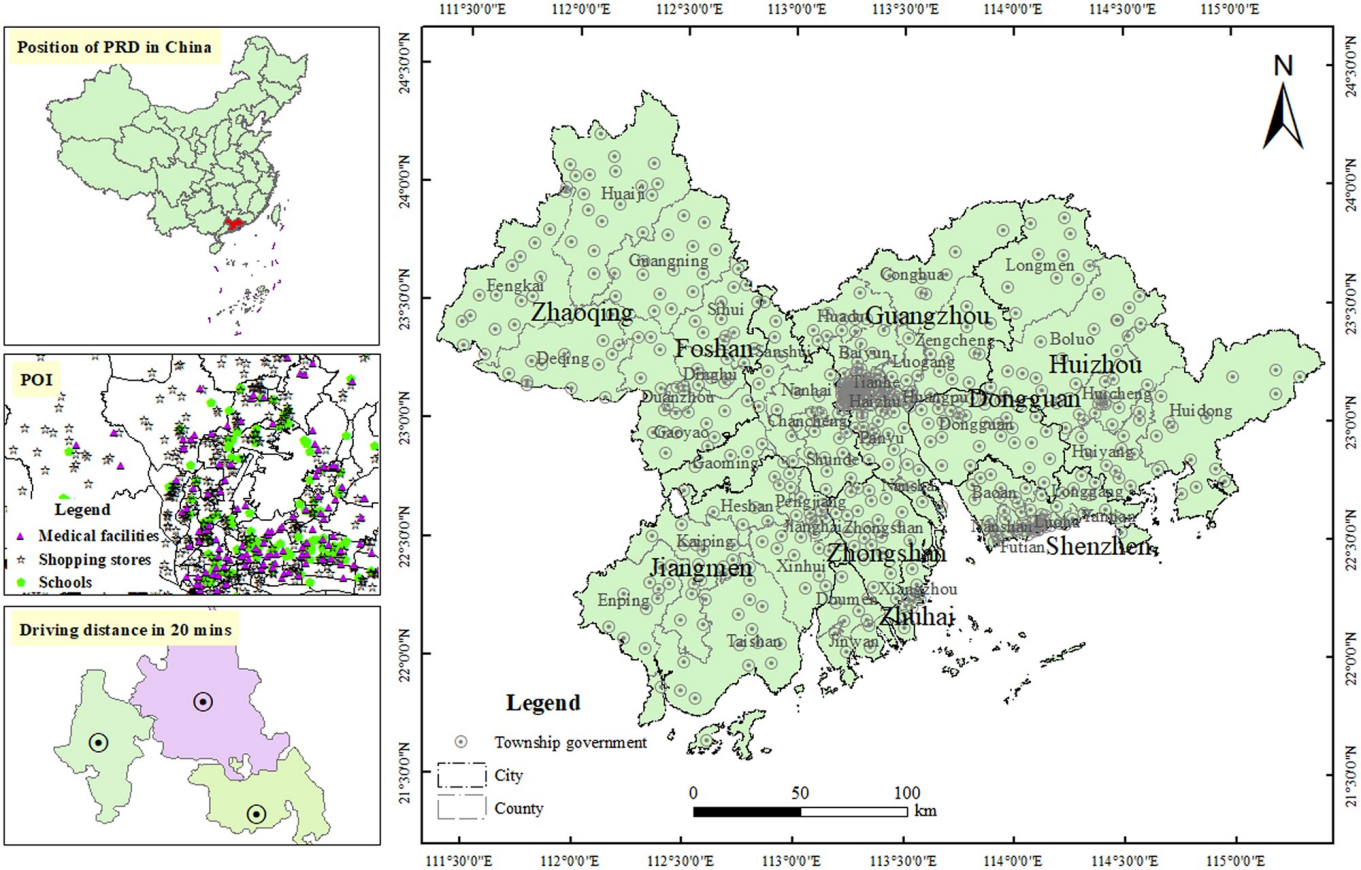
Research region and 20 min driving distance. Map created using ArcMap (version10.2) software from Esri (http://www.arcgis.com/).

The research region is urban agglomerations in the Pearl River Delta (PRD), which is located in the south–central part of Guangdong Province, China, and at the estuary of Pearl River, including the nine cities of Guangzhou, Shenzhen, Foshan, Zhuhai, Jiangmen, Zhongshan, Dongguan, Huizhou, and Zhaoqing (seen in Fig 1). With a total area of 54732.72 km^2^, the PRD is 30.47% of Guangdong Province. In 2015, the residential population of PRD was 58.7427 million, accounting for 54.15% of the Guangdong total, while regional GDP was 6.226778 trillion yuan, accounting for 76.62% of the Guangdong total.

Since the reform and opening up in 1978, with its good advantages in terms of location, the PRD quickly became one of the most developed areas in China. As urbanization and social economy develops at top speed, land use patterns are also undergoing dramatic changes. The overall characteristics of land use changes are dramatic changes in urban, industrial, transportation, and cultivated land. Land use changes in different regions are significantly different, and these changes have certain internal relations with industrialization, and the process and status of urbanization[41]. In addition, changes show obvious performance characteristics with regional differences[42]. The PRD, the largest city agglomeration in East Asia, optimizes land use patterns to balance development between production, human living standards, and ecology, improving the quality of urban spaces when industrial areas transform into urban areas.

### Data source and preprocessing

#### Land use data

Landsat imagery courtesy of NASA Goddard Space Flight Center and U.S. Geological Survey, downloaded from USGS (https://earthexplorer.usgs.gov/). Data in 1990 and 2000 were obtained by Landsat-5 TM, while data in 2015 by Landast-8 OIL, and data from all years are a mosaic of 7 images with a WRS-2 Path/Row of 123043, 123044, 123045, 122043, 122044, 122045, and 121044. By analyzing the characteristics of land use in the PRD, land is divided into six types, namely, cultivated land (CUL), forest land (FOR), grassland (GRA), waters (WAT), construction land (CON), and unutilized land (UNU). The classification accuracies of the three years (1990, 2000, and 2015) are 89.21%, 91.23% and 93.12%, having higher reliability, timeliness, and integrality.

#### Statistical data

These data mainly include demographic data and GDP data, all collected from Guangdong Statistical Yearbook of 1991, 2001 and 2016.

### Methods

#### Land use transfer matrix

This transfer matrix reflects information about the dynamic process during which land types change at a specific period in a certain area, data including land area type data at a specific time, and rich information about land type changes. The general formula of the land use transfer matrix is shown below[43]:

$$s_{ij}=\left[\begin{array}{cccc} s_{11} & s_{12} & \ldots & s_{1n}\\ s_{21} & s_{22} & \ldots & s_{2n}\\ \vdots & \vdots & \vdots & \vdots \\ s_{n1} & s_{n2} & \ldots & s_{nn} \end{array}\right]$$ (1)

where *s* is the area; *n* is the number of land use types before and after land transfer; *i* (*i* = 1, 2, …, *n*) is land use type before transfer; *j* (*j* = 1, 2, …, *n*) is land use type after transfer; and *s*_*ij*_ is the area of *j* when it is transferred from *i*. The elements in each line of the matrix represent information that *i* transfers to other land use types, and the elements in each column of the matrix represent the source information of *j* [44]. This matrix contains rich transformation information that land use types change during specific periods.

#### Spatial autocorrelation analysis

Spatial autocorrelation includes global spatial autocorrelation index and local spatial autocorrelation index. The spatial autocorrelation method has been widely used, for example, with regard to the environment[45, 46], land use and landscapes[30, 47–49], and carbon emissions[50–52]. Moran’s I index is used to measure global spatial autocorrelation, and the formula is as follows:

$$I=\frac{n\sum_{i}^{n}\sum_{j}^{n}w_{ij}(y_{i}-\bar{y})(y_{j}-\bar{y})}{(\sum_{i}^{n}\sum_{j}^{n}w_{ij})\sum_{i}^{n}{\left(y_{i}-\bar{y}\right)}^{2}}$$ (2)

where *n* is the total number of units in the study area, *y*_*i*_ and *y*_*j*_ are attribute values of points *i* and *j*, $\bar{y}$ is the average value of all attribute values in the study area, and *w*_*ij*_ is the spatial weight. Moran’s *I* is in the range of [–1, 1], indicating that there is negative correlation when the observed value is less than 0, an independent random distribution when it is equal to 0, and a positive correlation when it is greater than 0.

The local Moran’s *I*, proposed in 1995 by Anselin[53], is used to represent local spatial autocorrelation. The formula is as follows:

$$I=z_{i}\sum_{i}w_{ij}z_{j}$$ (3)

where *z*_*i*_ is the standard amount of mean value, *z*_*j*_ is the standardized quantity of the standard deviation, $z_{i}=\frac{x_{i}-\bar{x}}{\delta }$, and *δ* is the standard deviation of *x*_*i*_.

#### Principal component analysis

Principal component analysis (PCA) is an analytical and statistical method of converting multiple related elements into several uncorrelated comprehensive indicators. Under the principle of ensuring that the minimum amount of information is missing, PCA conducts dimensionality deduction to convert the many original indicators into less comprehensive indicators that can reflect the research, so that the study could improve its efficiency and accuracy. All demographic and GDP data which are required in PCA were collected from statistical yearbooks.

## Results

### Analysis on traffic accessibility based on internet map

The existing studies on traffic accessibility were conducted by establishing a spatial database based on GIS technology[54–56], but it would be hard to do research if there was no complete road network. With the development of Internet maps, detailed information, including road information about highway entrances and exits, road connections, and even community-road pavements, could be obtained. Since an Internet map could provide users with navigation services, it can better reflect real situations when calculating travel time and distance than if conducting a simulation study with GIS. Therefore, Internet maps were applied to research when traffic-accessibility analysis on townships is conducted. With an application programming interface (API), accessibility elements were based on medical facilities, shopping stores, and schools. The distance and time calculation from origin to destination on 4 February 2018. Considering the concept of hourly intercity traffic cycles, the study determined driving distance in 20 minutes (shown in Fig 1) at non-traffic-congestion hours, with the location of the township government as the center so as to maximally distinguish the differences of traffic accessibility in various townships.

Statistics for the number of medical facilities, shopping stores, and schools were found, and their data are from POI. There are three indicators to reflect traffic accessibility: first, the total number of facilities, including medical facilities, shopping stores, and schools, that can be reached within 20 minutes from the center of various townships, hereinafter referred to as ACC_NUMTOT; second, the total travel time needed from the center of various townships to the nearest medical facilities, shopping stores, and schools, hereinafter referred to as ACC_TIMTOT; third, the travel time needed from the center of a township to the center of a city to which the township is subordinate, hereinafter referred to as ACC_TIMCEN.

**Fig 2:**
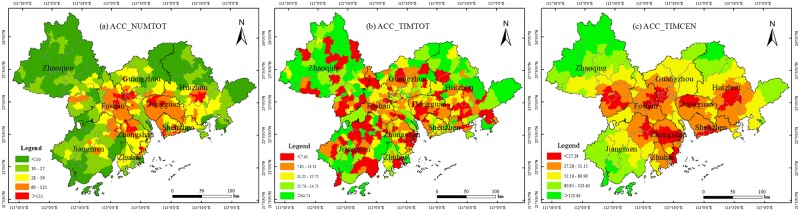
Distribution of traffic accessibility in the Pearl River Delta (PRD). Map created using ArcMap (version10.2) software from Esri (http://www.arcgis.com/).

By finding the statistics on the total number of medical facilities, shopping stores, and schools reachable within 20 min in each township, ACC_NUMTOT was drawn as seen in Fig 2a, where the traffic accessibility of various townships in PRD was analyzed. It was found that there is obvious spatial heterogeneity among the number of facilities reached within 20 min. This means that there are many townships in the central urban areas of Guangzhou, Foshan, Shenzhen, Zhongshan, and Huizhou, especially in the border area between Guangzhou and Foshan, where the number of townships showed that these are the areas that that had the most traffic accessibility. On the whole, the number of townships in the central urban area of Guangzhou, Foshan, Shenzhen, Dongguan, Huizhou, and Zhongshan was higher than that of surrounding areas like Zhaoqing, Jiangmen, and Huizhou.

When obtaining the coordinates of the nearest area with facilities in each township, the coordinates of origin (the location of the township government) and destination (the nearest location that has facilities) could be entered to calculate ACC_TIMTOT; results are shown in Fig 2b. Compared with the distribution feature of ACC_NUMTOT, the distribution feature of ACC_TIMTOT is equally distributed with fewer differences. As seen in Fig 2b, there is no obvious agglomeration center, and areas needing much more travel time are mainly concentrated in the remote townships of various regions, while travel time needed in central urban cities is less. On the whole, areas that need more ACC_TIMTOT are located in the peripheral areas in the PRD, while central areas in PRD, such as Guangzhou, Shenzhen, and Foshan, take less ACC_TIMTOT.

The formula mode of ACC_TIMCEN is the same as ACC_TIMTOT, and the results are shown in Fig 2c, where two characteristics can be seen: first, a feature of the layer structure in different cities is obvious, that is, the needed travel time is gradually increased from the center of the city; second, on the whole, ACC_TIMCEN is gradually increased from the center of Guangzhou, Foshan, Dongguan, and Shenzhen to the peripheral areas because road networks in urban center areas is developed, while there are only a few roads connecting the center with peripheral areas.

Through analysis, it can be found that traffic accessibility in the PRD is highly consistent with the economic-development level of each region. The fact that the number is higher in urban center areas and lower in peripheral areas is obvious. The spatial variation characteristics of ACC_NUMTOT are obvious too, since the value is higher in central areas such as Guangzhou and Foshan, while the value is lower in the peripheral areas of Zhaoqing, Jiangmen, and Huizhou. Differences of ACC_TIMTOT between different places are small, which indicates that the gap between the different townships to acquire services in education, medical treatments, and the market is small, and distribution justice could be guaranteed to the entire region. The characteristics of ACC_TIMCEN are the feature of the layer structure that regards respective cities as its center and the core area of PRD as its center.

### Global land use change analysis

**Fig 3:**
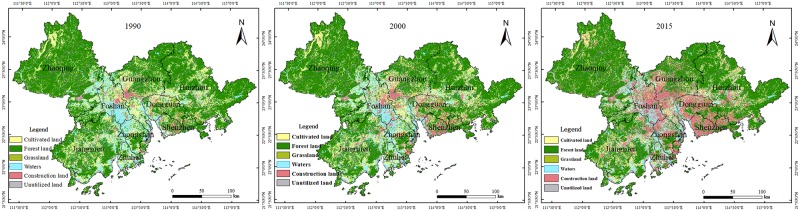
Distribution of land use in PRD. Map created using ArcMap (version10.2) software from Esri (http://www.arcgis.com/).

Land use data were obtained through Landsat data, and they are from the years 1990, 2000, and 2015. Land use types are divided into six parts, namely, CUL, FOR, GRA, WAT, CON, and UNU, as shown in Fig 3.

Statistics on the land use data of the PRD in the three years are shown in Table 1. By analyzing the table, it can be seen that land use types were mostly FOR and CUL in 1990 and 2000, while in 2015, they were mostly FOR and CON. As shown in the table, the proportion of FOR was over 50%, while the proportion of CUL was over 12% in the three years. During this period, the proportion of CON was gradually increased from 1.69% to over 16.67%, and the areas of GRA, WAT, and UNU did not change much. By utilizing GIS overlay analysis, the land use data of the PRD in 1990, 2000, and 2015 are shown in Table 2.

**Table 1:** Statistics of land use types in PRD (km^2^, %). Note: CUL, cultivated land; FOR, forest land; GRA, grassland; WAT, waters; CON, construction land; UNU, unutilized land.

Type	1990		2000		2015	
	Area	Proportion	Area	Proportion	Area	Proportion
CUL	10674.36	19.59	9942.18	18.17	6666.54	12.17
FOR	33328.28	61.17	32643.07	59.65	31215.38	57.00
GRA	876.68	1.61	844.94	1.54	670.09	1.22
WAT	7201.32	13.22	6869.66	12.55	5979	10.92
CON	1202.87	2.21	3217.11	5.88	9127.09	16.67
UNU	1197.05	2.2	1207.32	2.21	1103.25	2.01

**Table 2:** Land use transfer matrix of PRD from 1990 to 2015 (km^2^).

	**2015**	**CUL**	**FOR**	**GRA**	**WAT**	**CON**	**UNU**	**Total**
**1990**								
CUL		5931.78	537.40	30.40	140.46	5.08	12.98	6658.09
FOR		580.47	30273.10	53.21	231.80	12.71	39.62	31190.92
GRA		51.12	64.85	519.01	23.58	2.56	4.34	665.47
WAT		286.90	281.61	18.19	5337.16	8.58	25.46	5957.91
CON		3794.11	2101.44	250.48	1429.39	1171.71	165.58	8912.71
UNU		29.75	66.04	5.24	38.04	2.21	945.76	1087.04
Total		10674.12	33324.44	876.54	7200.43	1202.86	1193.75	54472.14

From the transfer-matrix analysis, it can be seen that land use changes from 1990 to 2015 were as follows: 31.15 km^2^ of CON was turned into other types, and 7741 km^2^, about 6.6 times more than CON in 1990, was turned into CON during the period, with the out-transfer area being 7709.85 km^2^ less than the in-transfer area. Of the CUL area, 4742.35 km^2^ was turned into other types, and an area of 726.31 km^2^ became CUL during this period, with the out-transfer area being 4016.03 km^2^ more than the in-transfer area. The CUL area was greatly reduced because 3794.11 km^2^ of CUL was turned into CON, accounting for 80% of the total area change of CUL. With regard to FOR, 3051.34 km^2^ was turned into other types, and an area of 917.82 km^2^ was turned into FOR during the period, with the out-transfer area being 2133.52 km^2^ more than the in-transfer. Among an area of 3051.34 km^2^, 2101.44 km^2^ area was transferred to CON, accounting for 68.87% of the total, and 537.4 km^2^ was transferred to CUL, accounting for 17.61% of the total. Of WAT, 1863.27 km^2^ was turned into other types, and 620.57 km^2^ was turned into WAT during the period, with the out-transfer area being 1242.52 km^2^ more than the in-transfer. In an area of 1863.27 km^2^, 1429.36 km^2^ was converted into CON, accounting for 76.71% of the total. Of GRA, 357.53 km^2^ was turned into other types, and 146.46 km^2^ was turned into GRA during the period, with the out-transfer area being 211.07 km^2^ more than the in-transfer. In an area of 357.53 km^2^, 250.48 km^2^ was converted into CON, accounting for 70.06% of the total. Of UNU, 247.99 km^2^ was turned into other types, and 141.28 km^2^ was turned into UNU during the period, with the out-transfer area being 106.71 km^2^ more than the in-transfer area. In an area of 247.99 km^2^, 165.58 km^2^ was converted into CON, accounting for 66.77% of the total.

As shown above, CON was gradually increased in the PRD from 1990 to 2015 since other types of land were turned into CON. The source of the CON increase is mainly the conversion of CUL and FOR. The conversion area of GRA and UNU was small, but it mainly turned into CON.

### Local construction land change analysis

**Fig 4:**
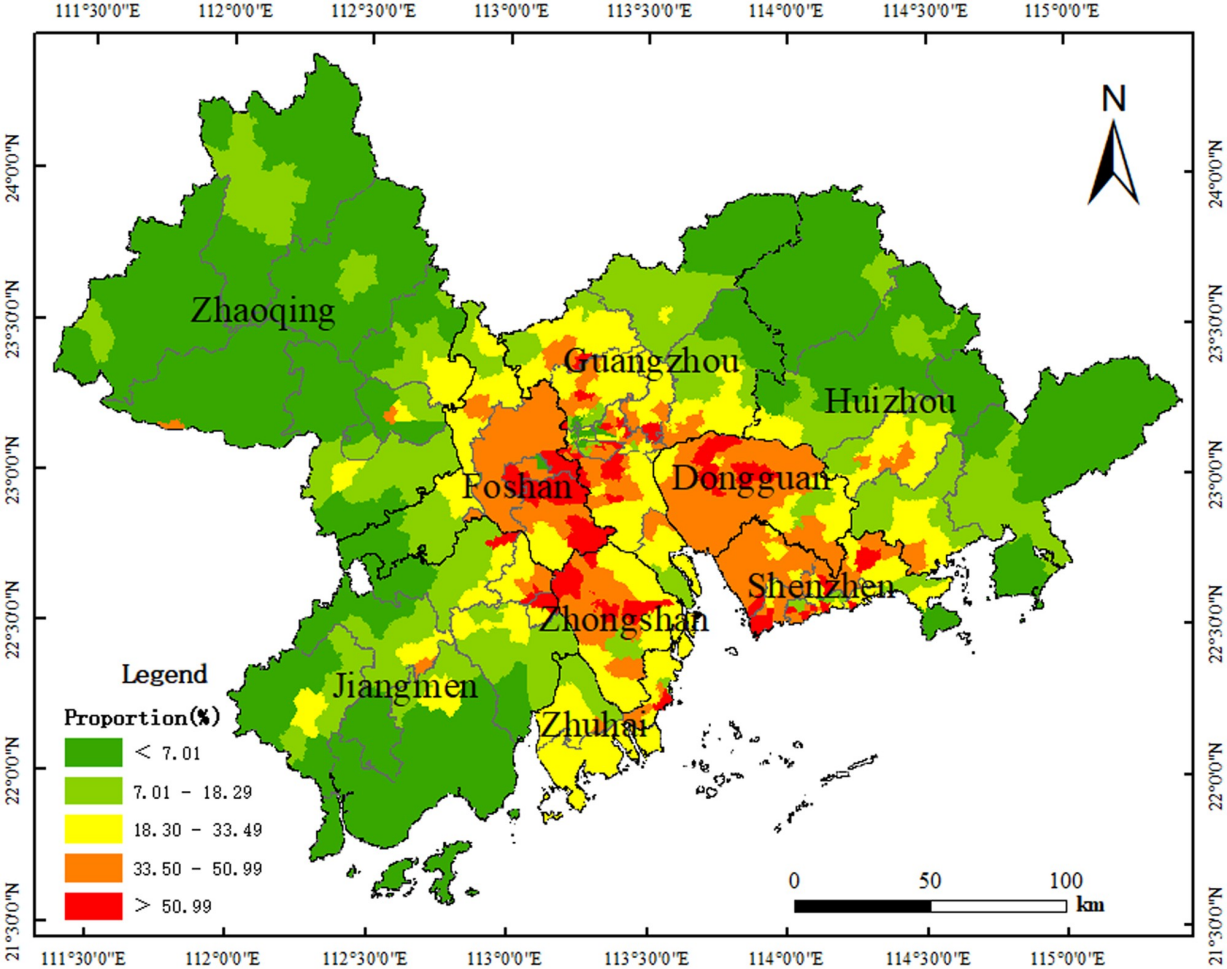
Proportion of the block transfer into construction land based on the township unit. Map created using ArcMap (version10.2) software from Esri (http://www.arcgis.com/).

The main characteristics of land use changes in the PRD from 1990 to 2015 are that CUL, GRA, and WAT are continuously converted into CON. With CON as the research object, statistics were analyzed to examine blocks that were converted into CON from 1990 to 2015, and to calculate the CON proportion of the total area of the township while regarding the township as the basic statistical unit; results are shown in Fig 4. The main characteristics of land use changes are other land converted into construction land, so it is very necessary to discuss construction land separately. With further analysis, it could be found that the township had a large proportion of blocks that were being converted into CON and were mainly distributed in Guangzhou, Foshan, Shenzhen, Dongguan, and Zhongshan. There are areas that were being converted into CON while having a larger proportion on the border of Guangzhou and Foshan, and on the border of Foshan and Zhongshan, while peripheral PRD cities like Zhaoqing and Huizhou have townships with smaller proportions of blocks that were being converted into CON. With Yuexiu district as its center, Guangzhou has an area with a low proportion value.

By selecting the global and local spatial autocorrelation index with the Geoda1.12 software, further research could be conducted; the spatial weight matrix was established with rook contiguity. Moran’s I was 0.69355, showing that there was positive correlation between CON changes, that is, townships with a higher proportion of blocks that were being converted into CON were adjacent to townships with a higher proportion as well, while townships with a smaller proportion of blocks that were being converted into CON were also adjacent to townships with a smaller proportion.

**Fig 5:**
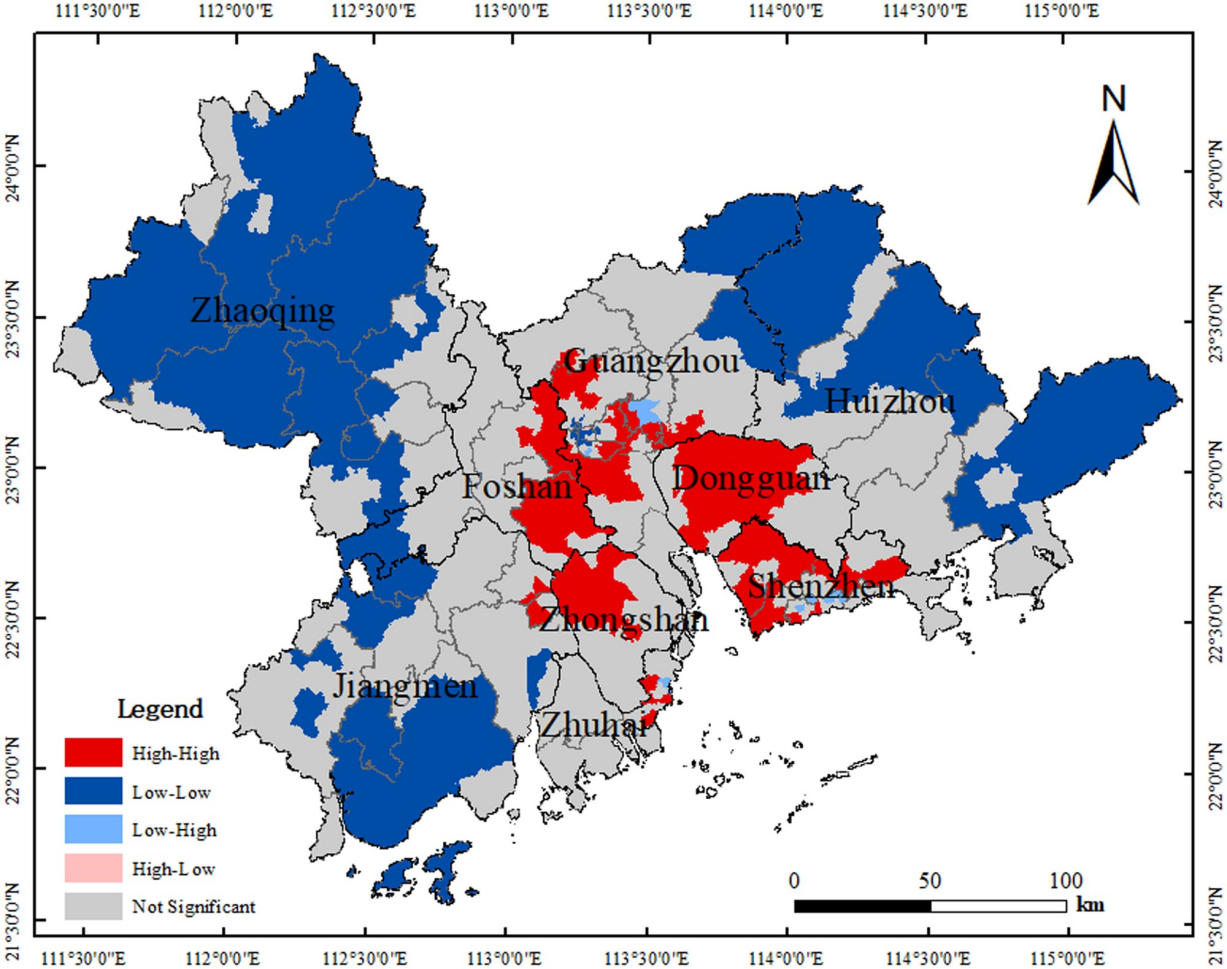
LISA of block transfer into construction land based on township unit. Map created using ArcMap (version10.2) software from Esri (http://www.arcgis.com/).

In order to find the spatial agglomeration area, local spatial autocorrelation should be analyzed as shown in Fig 5. After analysis, it could be seen that there are 293 townships belonging to the Not Significant type, with the largest quantity accounting for 50.43% of the total; this was followed by Low–Low and High–High types, with 163 townships belonging to Low–Low, accounting for 28.06% of the total, and 118 townships belonging to High–High, accounting for 20.31% of the total. Meanwhile, the number of Low–High and High–Low types was small, with 6 townships belonging to Low–High, accounting for 1.03% of the total, and 1 township belonging to High–Low, accounting for 0.17% of the total. Obviously, the increased area used for CON in the PRD during the period shows a layer-structure feature. The core areas of the Pearl River Estuary are dominated by the High–High type, while peripheral areas are dominated by Low–Low, and middle areas by Not Significant. To be specific, most townships in Dongguan belong to High–High, and Guangzhou had obvious Not Significant, High–High, and Low–Low features from the center to the peripheral areas. There are High–High areas on the border of Guangzhou and Foshan, Foshan and Zhongshan, Guangzhou, and Dongguan, Dongguan, and Shenzhen.

### Influence of traffic accessibility on land use and regional development

With 581 townships as the research unit, the study aimed to analyze the influence of traffic accessibility on land use and socioeconomic development in urban agglomerations. In the research, three variables were selected, that is, traffic accessibility, land use, and socioeconomic development. First, land use variables: the proportion of various land use types in 2015 is expressed as CLU_15, FOR_15, GRA_15, WAT_15, CON_15, and UNU_15, respectively; the proportion of changes made from 1990 to 2015 is found by using the proportion in 2015 minus the proportion in 1990, which is expressed as CLU15_90, FOR15_90, GRA15_90, WAT15_90, CON15_90, and UNU15_90, respectively. Second, traffic-accessibility variables are ACC_NUMTOT, ACC_TIMTOT, and ACC_TIMCEN. Third, socioeconomic development variables are two indicators, namely, density of population (DPOP) and per capita gross domestic product (PGDP).

**Fig 6:**
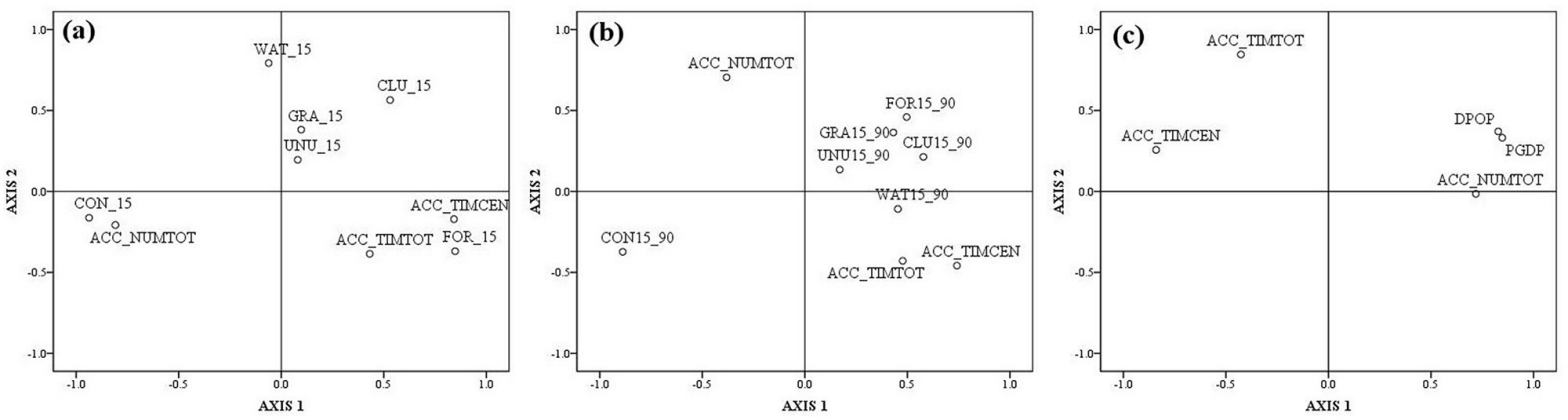
Scatter diagram of principal-component analysis.

Castella (2005) based on the analysis of land-use dynamics and regional development, summarized the influencing factors and used the Pearson correlation matrix and principal-component analysis to prove the impact of accessibility on land use and regional development[11]. So, using this method for reference, the article researched the influence of traffic accessibility on land use and regional development. First, analysis was conducted to study the influence of traffic accessibility on land use and socioeconomic development by using the Pearson correlation matrix. Then, principal variables were analyzed including traffic accessibility, proportion of various land types in 2015, proportion of changes made from 1990 to 2015, and socioeconomic development. One can regard the first principal component as the horizontal axis and the second principal component as the vertical axis to draw a scatter diagram for this research. The results of the Pearson correlation matrix are shown in Table 3, while the scatter diagram is shown in Fig 6. Fig 6a–6c are the scatter diagrams of principal-component analysis of traffic accessibility and the proportion of various land types in 2015, the proportion of land type changes made from 1990 to 2015, and socioeconomic development, respectively.

**Table 3:** Pearson correlation matrix.

	ACC_NUMTOT	ACC_TIMCEN	ACC_TIMTOT	CLU15_90	FOR15_90	GRA15_90	WAT15_90	CON15_90	UNU15_90	CLU_15	FOR_15	GRA_15	WAT_15	CON_15	UNU_15	PGDP
ACC_TIMCEN	–0.541**															
ACC_TIMTOT	–0.210**	0.460**														
CLU15_90	–0.02	0.274**	0.144**													
FOR15_90	0.019	0.239**	0.089*	0.023												
GRA15_90	–0.044	0.168**	0.072	0.113**	0.307**											
WAT15_90	–0.145**	0.228**	0.113**	0.088*	–0.033	0.038										
CON15_90	0.074	–0.425**	–0.200**	–0.692**	–0.551**	–0.361**	–0.500**									
UNU15_90	0.001	0.081	0.047	–0.026	0.104*	0.047	0.019	–0.161**								
CLU_15	–0.410**	0.370**	–0.008	–0.108**	0.297**	0.140**	0.210**	–0.202**	0.081							
FOR_15	–0.599**	0.674**	0.305**	0.419**	0.117**	0.147**	0.393**	–0.526**	0.071	0.174**						
GRA_15	–0.122**	0.059	–0.092*	–0.288**	–0.064	–0.113**	–0.074	0.265**	–0.043	0.124**	–0.026					
WAT_15	–0.099*	–0.150**	–0.090*	–0.218**	0.183**	0.103*	–0.379**	0.201**	0.04	0.249**	–0.373**	0.041				
CON_15	0.738**	–0.684**	–0.255**	–0.269**	–0.249**	–0.209**	–0.273**	0.449**	–0.057	–0.552**	–0.835**	–0.085*	–0.116**			
UNU_15	–0.128**	0.012	0.095*	0.045	–0.113**	–0.032	–0.058	0.104*	–0.400**	–0.026	–0.047	0.102*	0.07	–0.096*		
PGDP	0.448**	–0.584**	–0.150**	–0.115**	–0.396**	–0.225**	–0.144**	0.380**	–0.153**	–0.493**	–0.564**	–0.264**	–0.066	0.701**	0.060	
DPOP	0.453**	–0.530**	–0.129**	–0.118**	–0.358**	–0.392**	–0.226**	0.406**	–0.032	–0.498**	–0.533**	–0.142**	–0.139**	0.704**	–0.011	0.791**

** representation correlation is significant at the 0.01 level (two-tailed);

* representation, 0.05 level.

As shown in Fig 6a, the first two axes account for 55.13% of the total variance, with Axis 1 accounting for 38.31% and Axis 2 for 16.82%. In Fig 6b, the first two axes account for 46.22% of the total variance, with Axis 1 accounting for 30.14% and Axis 2 for 16.08%. In Fig 6c, the first two axes account for 76.72% of the total variance, with Axis 1 accounting for 56.16% and Axis 2 for 20.56%. By analyzing the influence of traffic accessibility on land use through Fig 6a and the Pearson correlation matrix, it can be seen that: ACC_NUMTOT is positively correlated with the proportion of CON on Axes 1 and 2, and is negatively correlated with the proportion of CUL, UNU, and GRA; ACC_NUMTOT is negatively correlated with the proportion of FOR on Axis 1 and negatively correlated with the proportion of WAT on Axis 2. ACC_TIMCEN is negatively correlated with the proportion of WAT on Axes 1 and 2, and positively correlated with the proportion of FOR; on Axis 1, it is negatively correlated with the proportion of CON, and positively correlated with the proportion of CUL and FOR. ACC_TIMTOT is negatively correlated with the proportion of WAT on Axes 1 and 2, and positively correlated with the proportion of FOR; on Axis 1, it is negatively correlated with the proportion of CON, and positively correlated with the proportion of FOR and UNU; on Axis 2, it is negatively correlated with the proportion of GRA. Quadrant distribution in the scatter diagram shows that: the distribution features of ACC_NUMTOT are the same as the distribution features of CON; the distribution features of ACC_TIMTOT and ACC_TIMCEN are the same as the features of FOR; and the distribution features of CUL, GRA, and UNU are same. The influence of traffic accessibility on land use is mainly reflected in that there is more CON but less CUL and FOR distribution in townships with higher traffic accessibility (townships with a higher ACC_NUMTOT, and low ACC_TIMTOT and ACC_TIMCEN), and it also reflects that the influence of traffic accessibility on water areas is significantly different.

By analyzing the influence of traffic accessibility on land use through Fig 6b and the Pearson correlation matrix, it can be seen that ACC_NUMTOT is negatively correlated with the changes of WAT on Axes 1 and 2, and it has no obvious correlation with the changes of CON, showing that the influence of ACC_NUMTOT on land use is weak. ACC_TIMCEN is positively correlated with the changes of WAT on Axes 1 and 2, negatively correlated with the changes of CON on Axis 1, and positively correlated with the changes of CUL, FOR, and GRA, indicating that if townships are far away from urban centers, the proportion of CON decreases, and the proportion of CUL, FOR, GRA, and WAT would also decrease; if townships are near urban centers, the proportion of CON increases, and the proportion of CUL, FOR, GRA, and WAT would also increase. The distribution features of ACC_TIMTOT are similar with ACC_TIMCEN. ACC_TIMTOT is positively correlated with the changes of WAT on Axes 1 and 2, negatively correlated with the changes of CON on Axis 1, and positively correlated with the changes of CUL and FOR. This shows that, if people take longer to reach the nearest facilities, the increasing proportion of CON, and the decreasing proportion of CUL, FOR, and WAT would be smaller in townships with relatively poor transportation, and that, if people take less time to reach the nearest facilities, the increasing proportion of CON, and the decreasing proportion of CUL, FOR, and WAT would be larger in townships with relatively developed transportation. ACC_TIMTOT and ACC_TIMCEN are significantly related to land use changes. Townships with more facilities and that take less time to facility locations or urban centers could have more CON that is converted from FOR and CUL areas, while townships with fewer facilities and that take more time to facility locations or urban centers are less likely to have land use changes. The correlation between ACC_NUMTOT and land use changes is weak, indicating that the influence of the number of facilities on land use changes is weak due to a comprehensive, reasonable, and fair infrastructure layout in highly developed urban agglomerations. It is also noticeable that ACC_TIMTOT, ACC_TIMCEN, and ACC_NUMTOT could promote conversion from WAT to CON in urban agglomerations in PRD with a developed river network. And the development of infrastructures has a significant impact on the river systems in PRD. By analyzing that scatter diagram, it can be found that CON changes are in the opposite direction from changes of CUL, FOR, UNU, and GRA, and this is CON’s most significant difference with FOR and CUL. There are more changes of CON in townships with higher traffic accessibility (townships with higher ACC_NUMTOT, and low ACC_TIMTOT and ACC_TIMCEN), and the influence of ACC_TIMCEN on CON changes is maximal, showing that distance from a city has become the most important driving force for other types of land turning into CON.

By analyzing the influence of traffic accessibility on regional development through Fig 6c and the Pearson correlation matrix, it can be seen that ACC_NUMTOT is positively correlated with DPOP and PGDP, and ACC_TIMTOT and ACC_TIMCEN are negatively correlated with DPOP and PGDP, on Axis 1, indicating that townships with higher traffic accessibility (townships with higher ACC_NUMTOT, and low ACC_TIMTOT and ACC_TIMCEN) would have higher DPOP and PGDP, while townships with low traffic accessibility (townships with low ACC_NUMTOT, and higher ACC_TIMTOT and ACC_TIMCEN) would have lower DPOP and PGDP, which proves that traffic accessibility could promote socioeconomic development. As shown in the scatter diagram, DPOP and PGDP are distributed almost in the same point of the quadrant, showing that the distribution feature of DPOP is similar to that of PGDP.

## Discussion

**(1) Concentrated on township units and used new methods.** Compared with related research, such as Zhang [9], Reimets[36] and Castella[11], the study concentrated on smaller township units and used new research methods, such as Internet maps and POI.

**(2) Limitations.** In this study, the rate of changes in the use of agricultural and forest land for other purposes depends on legal provisions regulating the process of their protection, as well as spatial planning documents that provide for comprehensive development and land use; this study considered these parameters less. Moreover, due to data limitations, it would be difficult to obtain past information about traffic accessibility by using Internet maps. Thus, in the following studies, it should be noted that such information is needed for the gathering of daily data, and that the studies should be conducted by combining Internet maps and past road-network data, and by considering spatial planning documents.

## Conclusion

Based on the analyses on traffic accessibility and land use changes in the PRD, we studied the influence of traffic accessibility on land use and socioeconomic development with the Pearson correlation matrix and principal-component analysis. The results are summarized as follows:

1. In general, the ACC_NUMTOT of the central cities in Guangzhou, Foshan, Shenzhen, Dongguan, Huizhou, and Zhongshan was higher, while in peripheral areas like Zhaoqing, Jiangmen and Huizhou, the value was lower. Differences in ACC_TIMTOT between the areas were small and equally distributed in value. There was no obvious regional agglomeration center when analyzing ACC_TIMTOT, which indicates that differences in obtaining education, medical treatment, and market services are small, and overall fairness is high in townships in PRD, though there are still differences. ACC_TIMCEN reflects two distinct features of a layer structure: one is that the travel time needed from the center to go out gradually increases in various cities, and the other is that the travel time needed from core areas like Guangzhou, Foshan Dongguan, and Shenzhen to peripheral areas gradually increases as a whole.
2. The most obvious feature of land use changes is that CUL, FOR, and WAT areas are turning into CON. With spatial autocorrelation analysis, it was found that there was positive spatial autocorrelation in CON areas that converted from other land types; core areas of the Pearl River Estuary were dominated by the High–High type, while peripheral areas by the Low–Low type, and middle areas by the Not Significant type. There are also High–High areas on the border of Guangzhou and Foshan, Foshan and Zhongshan, Guangzhou and Dongguan, and Dongguan and Shenzhen.
3. Traffic accessibility has significant impacts on land use and socioeconomic development. The influence of traffic accessibility on land use is reflected in that townships with developed transportation exhibit a higher proportion of CON and lower proportion of CUL and FOR. Such influence is very complicated, as FOR and CUL areas are more likely to turn into CON in townships with low ACC_TIMCEN and ACC_TIMTOT, and FOR and CUL areas are less likely to turn into CON in townships with higher ACC_TIMTOT and ACC_TIMCEN. ACC_NUMTOT has a weak impact on land use changes. Townships with developed transportation have higher values in DPOP and PGDP, while townships with poor transportation have lower values in DPOP and PGDP, which indicates traffic accessibility could promote socioeconomic development.
4. ACC_TIMTOT, ACC_TIMCEN, and ACC_NUMTOT could influence the conversion of land type from WAT to CON in PRD with a developed river network. The construction of traffic facilities impacts the reduction of WAT.

## Supporting information

S1 Table Administrative list of PRD. (XLSX)
